# Mechanisms causing the transition between spatial pattern long transients

**DOI:** 10.1098/rsos.251754

**Published:** 2025-10-29

**Authors:** Linhao Xu, Donald L. DeAngelis

**Affiliations:** ^1^ Environmental Science and Policy, University of California Davis, Davis, CA, USA; ^2^ Department of Biology, University of Miami, Coral Gables, FL, USA

**Keywords:** spatial pattern, travelling wave, intransitive network, lattice model, local dispersal, stage structure

## Abstract

Regular self-organized spatial patterns can be observed in many ecological systems. Some such patterns are quasi-stable; that is, they can switch to a different spatial pattern on a relatively short time scale in the absence of external changes in environmental conditions, aside from minor stochastic events. They are referred to as long transients. Although long transients have been studied mathematically, the detailed mechanisms by which a pattern can suddenly switch in nature are not well understood. Here we study, through spatial simulation of spatial patterns of an empirically based model, a type of intransitive loop plane travelling wave, which can switch to different spatial patterns through minor events. Close study of the simulations allows the causal chains involved in the switch to be determined in precise ecological detail by focusing on local interactions. In particular, this indicates that in real ecological systems, even though they may be resilient over long time periods, there can be vulnerabilities. These vulnerabilities include time lags in some interactions, which even small perturbations can eventually expose, leading to instabilities changing the spatial pattern. We show that patterns from intransitive loops are especially susceptible to such instabilities. The results are applicable to ecological systems of interest.

## 1. Introduction

Self-organized spatial patterns have been observed in many ecosystems [1–6]. Reaction–diffusion partial differential equation models have been successful in describing these in terms of feedback relations between organisms and environmental conditions such as water [7–10]; e.g. alternating patterns of vegetation and bare soil in drylands. The emergence of such patterns has been credited with contributing to maintaining resilience under environmental stress [11].

Sudden changes in such systems, from one type of spatial pattern to a different one, have also been observed. Such switches can occur rapidly in systems that potentially have more than one potential state [12,13]. These switches can result from gradual changes in an environmental variable external to the system; for example, a semi-arid ecosystem consisting of patches of vegetation spaces can switch rapidly to only bare soil with slight decreases in rainfall [14–16]. If such a system also exhibits hysteresis, in which conversion back to patchy vegetation may require a higher rate of precipitation than the rate below which the switch to bare soil occurred, the sudden change is mathematically termed a critical transition. Mathematically, Turing instability is often used to explain the emergence of patches of vegetation under a gradual increase in precipitation from combinations of short-distance positive feedback and long-distance negative feedback on water availability [17].

However, other switches between states can occur even in the absence of changes in external conditions. In these cases, the original state may be only quasi-stable. These quasi-stable states are also referred to as ‘long transients’ when the state persists for a long time before rapidly switching to another state. Many empirical examples of long transients have been identified in a variety of ecosystems: benthic marine systems [18], coral reefs [19], plant–herbivore systems [20], dryland ecosystems [21] and Dungeness crab population dynamics [22], among others; see [23] for a review.

Investigating the causal pathways that can lead to drastic switches in patterns that have persisted over a long period of time is an active area of inquiry [23,24]. Recent reviews of progress on long transients have been published [25,26]. Because of the unexpectedness of their end, long transient ecological systems have implications for conservation, as apparently stable states may be transient, causing issues for management [27,28]. Long transient spatial patterns have been explained in terms of such mathematical phenomena as ‘ghost equilibria’ and ‘crawl by’ trajectories close to saddle points, as well as stochastic perturbations that knock a system into a different state [24].

Understanding such switches between spatial patterns in the absence of external change may also be aided by considering mechanisms besides Turing instabilities. Alternative explanations have been offered for some spatial patterns. For example, [29] showed that the physical theory of phase separation, based on density-dependent movement, could explain many spatial patterns. Another mechanism by which spatial patterns may emerge is that of intransitive loops. The well-known rock–scissors–paper interactions are examples of intransitive loops and usually described as competition networks in which species A is dominant over species B, which is dominant over species C, which is dominant over species A. This can result in coexistence of all three in temporally and spatially varying patterns. The occurrence of such systems is now established for marine sessile organisms [30] and there is an extensive theory and modelling literature [31–43].

As we will argue, intransitive loop patterns are vulnerable to being long transients when subject to even very week perturbations; thus whether some observed spatial patterns are due to intransitive loops is a relevant question. In that regard, it is important to note that intransitive networks can arise in other ways than through three-species competition. They can occur in systems in which there are at least three states that can alternate in an intransitive fashion. Descriptions of intransitive networks in ecological systems have been given by [44–46]. A particular case is that of two competitors, one of which is dominant over the other, which it would gradually exclude, but which has a natural enemy that can exert strong enough regulation on the dominant competitor to prevent the subdominant competitor from being excluded. This system is intransitive, as the dominant competitor would eliminate the inferior one, but the natural enemy can invade the dominant competitor and weaken it such that the subdominant competitor, if it still exists in the system, can reinvade, causing the natural enemy to then decline to a low level.

When the element of spatial mobility is included for the species, all three can coexist. This was shown by [46] for a cellular automata model for two competing ant species, one dominant and one subdominant and a phorid fly that is parasitic of the dominant ant species. The authors interpreted the system as partly a Turing and partly a rock–scissors–paper interaction. That model, based on observations of the species in Puerto Rico, showed that both clumped patterns of the species as well as spiral-like patterns emerge. This gives rise to spiral spatial patterns. Another example that could be interpreted as an intransitive network is the spiral spatial pattern that has been observed in high-altitude wetlands [47]. Three states, rain-stimulated vegetation, followed by the presence of herbivores that kill local grass but that stimulate new growth of grass in the neighbourhood, create a growing spiral pattern.

The case of two competing species in which there is a natural enemy of one is of special interest, as it is a classic motif in ecology. Here we contribute to the general understanding of the emergence of spatial structure in such a system through its behaviour as an intransitive loop, as well as how it can end as a long transient. In previous work we showed that a two-dimensional plane travelling wave could emerge, which, after a long period of time, ended as a transient in a chaotic pattern [48]. In [49] is reported a detailed study of why the end of the long transient occurred at a particular time. In that study, results were produced using a detailed and thoroughly parametrized spatial model that included spatial movement and life cycle of the natural enemy, so that the detailed dynamics could be examined.

Here we further study the system of two competitors and the specialist natural enemy. We produce a new result that the plane travelling wave created by an intransitive network can switch to a spiral wave under a perturbation and the spiral wave can undergo further long-transient behaviours. But our main purpose here is to integrate the modelling results with broader understanding of spatial pattern and long transient behaviour. We use a modification of the model used earlier, because it is well parametrized, but we argue that its main features are quite general. The system is simulated on a 50 × 50 lattice of 1 × 1 m^2^ pixels with equations for each species in all spatial cells (pixels) on daily time steps. An important feature is that on the lattice boundaries the natural enemy is restricted, to allow at least a small population of the dominant competitor to persist. This model had the original purpose of simulating effects of experiments on biocontrol of an invasive floating macrophyte competing with submersed vegetation in a natural setting (Lake Okeechobee, Florida), though here the theoretical aspects are of interest.

## 2. Material and methods

The model analysed here is first explained in terms of its general characteristics of a rock–scissors–paper system that is conducive to formation of a two-dimensional travelling wave, as well as causing the pattern to be quasi-stable and thus vulnerable to ending as a long transient.

### 2.1. General model characteristics

#### 2.1.1. Species interactions

There are two competing species, one of which is a dominant competitor that can exclude the subdominant competitor in the absence of other factors. A third species is a specialist natural enemy of the dominant competitor and can regulate that competitor to a low enough population density that it cannot exclude the subdominant competitor. The natural enemy does not affect the subdominant competitor and cannot exist on its presence alone.

#### 2.1.2. Spatial setting

The species occupy a two-dimensional lattice in which the equations for all three species occur on each pixel. Species populations can be driven to low levels on pixels but tendency towards extinction is a negative exponential, so that remnant populations can persist for some time. This can be pictured as a 50 × 50 m^2^ area with two competing vegetation species, one of which has a natural enemy with a life cycle of several stages.

#### 2.1.3. Movement rules

Each species can move, with all movements being local and only to adjacent pixels and also depending on densities on the donor and recipient pixels. Therefore, movement rates are relatively slow, as is generally necessary for establishment of travelling waves from intransitive systems.

#### 2.1.4. Boundary conditions

We have used non-periodic reflecting zero-flux boundary conditions and limited the effect of the natural enemy on the dominant competitor on the boundary pixels, to lessen the possibility of complete extinction of at least small populations of species on edges. Although these boundary conditions do not seem indispensable for creating plane travelling waves ([50] and [51] produced plane travelling waves with periodic boundary conditions), the boundary conditions in our model facilitated emergence of plane waves.

#### 2.1.5. Time lags

Time lags must exist in the type of travelling wave system being considered here. In particular, a lag in time in the response of the natural enemy to the dominant competitor is necessary for the travelling wave to emerge, as it allows that competitor to stay spatially ahead of the natural enemy to escape being driven to extremely low values. Thus, the natural enemy is assumed not to have complete control of the dominant competitor but to have enough control to prevent it from excluding the subdominant competitor. At the same time, the time lag can pose a vulnerability to an established spatial pattern. If the time lags in the system are large enough, even a small disturbance to the spatial pattern might create a situation in which the natural enemy loses the limited control that it has on the dominant competitor, such that instability in the pattern can occur and grow. Two types of time lags that can occur are the following. One is the time it takes for movements of the natural enemy from one pixel to another, which may be limited to local movement steps. That lag can limit the ability of the natural enemy to completely overtake the dominant competitor spatially. The second is the time lag in the life cycle of the natural enemy, such as time between reproduction and older life stages that are the main consumers. That lag can delay the effect of the natural enemy on the dominant competitor.

#### 2.1.6. Stochasticity

Stochastic events can be endogenous to the system in terms of probabilities of movement among adjacent pixels, which can be partly random. In addition, stochastic events may be imposed externally, ranging from very small to large. We demonstrate in a specific model that even very small perturbations may trigger instability and change in spatial pattern.

**Figure 1.**
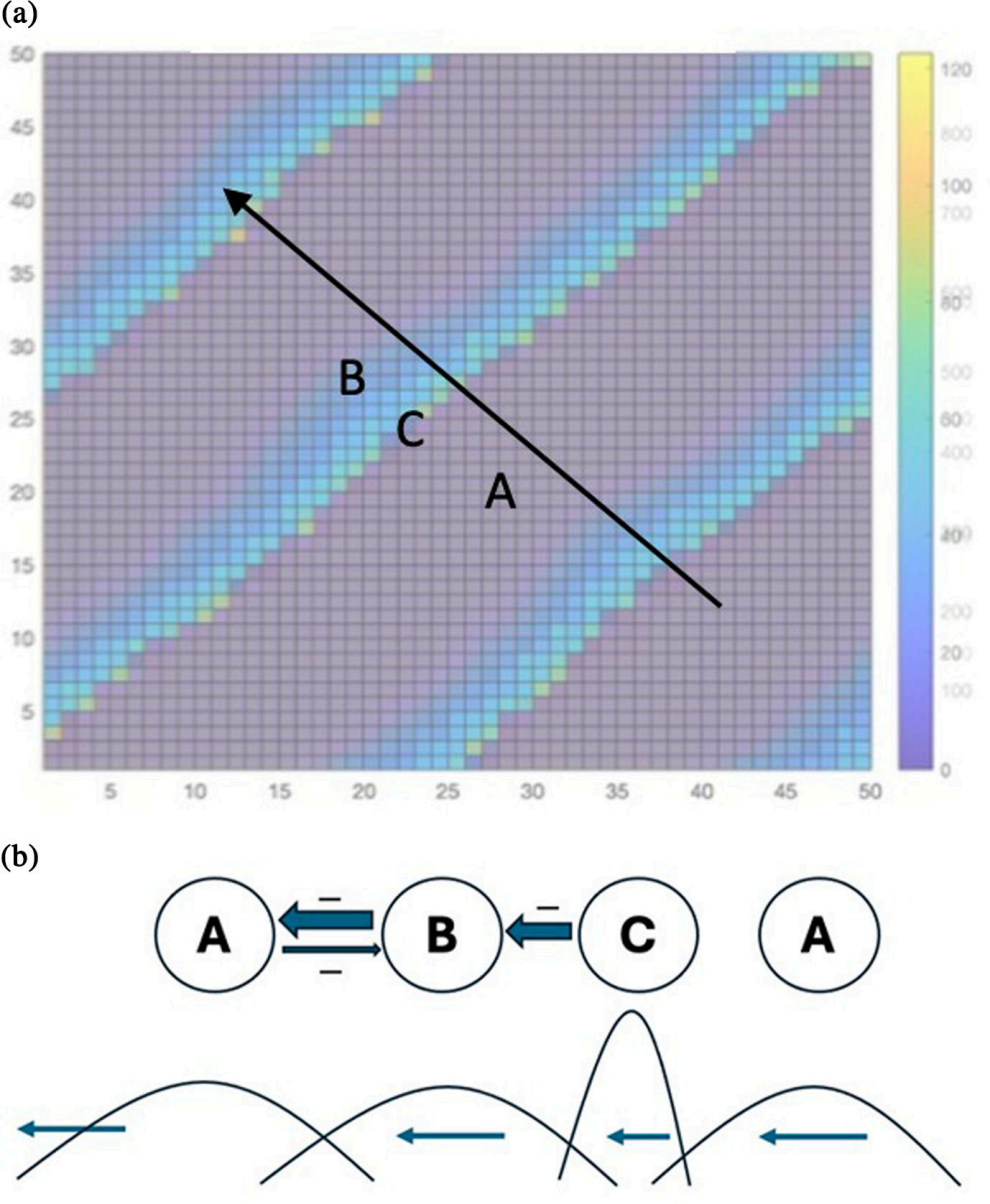
(a) Snapshot of hypothetical travelling wave moving towards the upper left; subdominant competitor (A, grey stripe), dominant competitor (B, green stripe) and natural enemy (C, very thin yellow pixels at trailing edge of the dominant competitor stripe at which the maximum levels of the natural enemy population occur). The subdominant competitor (A) follows the natural enemy. (b) Schematic diagram of the pattern of the travelling wave.

The above conditions appear to be important ingredients in allowing a travelling wave to emerge in an ecological model. Figure 1 summarizes the interactions. The travelling wave that can emerge from the system’s attributes described above could be stable, but it will be shown that the time lags can also be vulnerabilities that make it quasi-stable and thus possibly ending at some time.

### 2.2. A specific model

A specific model with the above general characteristics was developed by [48] for application to biocontrol of an invasive species. Similar to the model of [46], there are two competitors, dominant and subdominant, and a natural enemy, a weevil, of the dominant species. The species involved in the model in [48] are two competing aquatic macrophytes, one a submersed macrophyte (SAV) and the other an invasive floating macrophyte (FAV), and the third a natural enemy of the floating macrophyte, which is the dominant competitor. The model of competition between FAV and SAV is based on the equations of [52], used here in discrete-time form with daily time steps:


(2.1) $$S\left(t+1\right)=S\left(t\right)+\;r_{s}S\left(t\right)\frac{n\left(t\right)}{n\left(t\right)+h_{s}}\frac{1}{1+a_{s}S\left(t\right)+bF\left(t\right)}-l_{s}S\left(t\right),$$

(2.2) $$F\left(t+1\right)=F\left(t\right)+\;r_{f}F\left(t\right)\frac{n\left(t\right)}{n\left(t\right)+h_{f}}\frac{1}{1+a_{f}F\left(t\right)}-l_{f}F\left(t\right)-F_{consumed},$$

where *S* and *F* are dry weight biomasses (g dW m^−2^), *r_s_* and *r_f_* are the maximum growth rates (day^−1^), and *l_s_* and *l_f_* are the loss rates (day^−1^) from respiration and mortality, of SAV and FAV, respectively. $F_{consumed}$ is the consumption rate of weevil larvae on the FAV (g dW m^−2^ day^−1^). In this model, competition of the two species is exploitative, for a limiting nutrient, *n*. Nutrient concentration, *n*, in soluble form in the water column in mg l^−1^, is given by *n*, and *h_s_* and *h_f_* are the half-saturation values for nutrient uptake of SAV and FAV. Parameters *a_s_* and *a_f_* represent intraspecific competitive effects of SAV and FAV, respectively, and *b* is the effect of shading of the FAV on the SAV. It is assumed that total nutrient in the system, *N*, is fixed in the system, and it is divided among the vegetation and soluble form in the water column, as in the model of [52]:


(2.3) $$N=\;n+nq_{s}S+nq_{f}F,$$

where *q_s_* and *q_f_* are coefficients representing the fractions of nutrient tied up in the vegetation per unit dry weight biomass. Variables and parameters are defined and the latter given values in electronic supplementary material, tables S1 and S2.

This model simulates how biocontrol on an invasive FAV will affect the FAV–SAV interaction. To do this a biocontrol agent is added to the model. The modelling of the biocontrol agent model is based on the [53] model of the interaction of the invasive plant water hyacinth (*Pontederia crassipes*) and the weevil *Neochetina eichhorniae*. In [53], differential equations were used for the invasive plant biomass and life stages of the weevil, adults and first- and second-stage larvae. But the model used here is modified to discrete-time equations, such that the life cycle of the weevil could be described by a Leslie matrix. The Leslie matrix equation for our model of the weevil life stages is


(2.4) $$M\;=\left|\begin{array}{cc} \begin{array}{cc} E_{12,12} & 0\\ T_{E,L1} & L1_{29,29} \end{array} & \;\;\;\;\begin{array}{cc} \;\;\;0\;\;\;\; & \;\;\;\;0\\ 0 & \;\;\;\;0 \end{array}\;\\ \begin{array}{cc} 0 & \;\;\;\;\;\;\;T_{L1,L2}\\ 0 & 0 \end{array} & \;\;\;\;\;\begin{array}{cc} L2_{14,14} & 0\\ T_{L2,P} & P_{20.20} \end{array} \end{array}\right|\;,$$

where *E*_12,12_ is a 12 × 12 submatrix describing the egg stage on a daily basis, and *L*1_29,29_, *L*2_14,14_ and *P*_20,20_ are submatrices for two larval stages and the pupal stage. The off-diagonal *T* submatrices represent transitions between stages. The full Leslie matrix is in [48], which includes a description of the effects of density dependence mortality on the L1 larval stage and the consumption rate, *F_consumption_*. There is a separate differential equation for the adults, lumped together as a single variable in each pixel:


(2.5) $$A\left(t+1\right)=\frac{S_{A}A\left(t\right)}{1+\beta A\left(t\right)/F\left(t\right)}+S_{P}Pupae_{20}\left(t\right),$$

where *S_A_* is maximum daily survival. The second-stage larvae feed on the invasive plant, while the first-stage larvae experience density-dependent mortality, in the form of the ratio of larval biomass to the FAV biomass. The life cycle model is described in more detail in electronic supplementary material, appendix 2, and variables are defined in electronic supplementary material, table S3.

The difference equation model described above for SAV, FAV and the five stages of the weevil life cycle is implemented in each cell or pixel of a 50 × 50 spatially explicit lattice model of 1 m × 1 m pixels, with each variable having a value in each pixel. This is similar to the extension of the [52] model to a lattice by [54], except that the current model includes the biocontrol weevil. The SAV–FAV competition was parametrized from data of [52] and [54], while the weevil life cycle was parametrized from [53] (see also [55]). All these parameters are shown in electronic supplementary material, table S4. The model is fully described in [48], in which a schematic of the interactions is shown.

Both the SAV and FAV were assumed to spread vegetatively from spatial pixel to adjacent spatial pixel. The adult weevils could also spread from pixel to pixel. The spread of both vegetation types and the adult weevils was partly stochastic. The rules for the dispersal of each are described in appendix 1. Diffusion of the nutrient, *n*, in the water column is also included in the model.

### 2.3. Initial conditions, boundary conditions and stochastic perturbations

In the model, initial conditions could be set in a variety of ways. Each species could be initiated with any starting biomasses in any of the pixels. Simulations started by randomly establishing initial amounts of SAV and FAV on the 50 × 50 cell lattice. A small number of adult biocontrol insects were added 600 days after the start of the simulation, and then periodically every 300 days. Adults were added to cells with FAV in centre region of the plot; that is, in the region of (18 ≤ *i* ≤ 32, 18 ≤ *j* ≤ 32). Two adults were added with 25% probability to cells with FAV biomass greater than a threshold value *F_threshold_* of 20 g dW m^−2^. Details on these additions of adults are provided in electronic supplementary material, table S5. On day 13 000 of the simulation, it was assumed that environmental conditions changed such that the consumption rate per larva on the FAV—in terms of the damage to the FAV biomass, though not affecting larval survival—increased (see electronic supplementary material, table S4). Non-periodic reflecting boundary conditions were imposed on the end pixels of sides of the lattice, such that the adult weevils could not move to FAV on those pixels. Although SAV could compete with FAV on those pixels, that condition increased the likelihood that FAV would not go to extinction on the lattice. This was designed to represent biocontrol of invasive FAV experiments in Lake Okeechobee in which square areas were treated with biocontrol insects, but invasion of FAV from the perimeter could still occur (Melissa Smith 2024, USDA, personal communication).

## 3. Results

### 3.1. Overview

**Figure 2.**
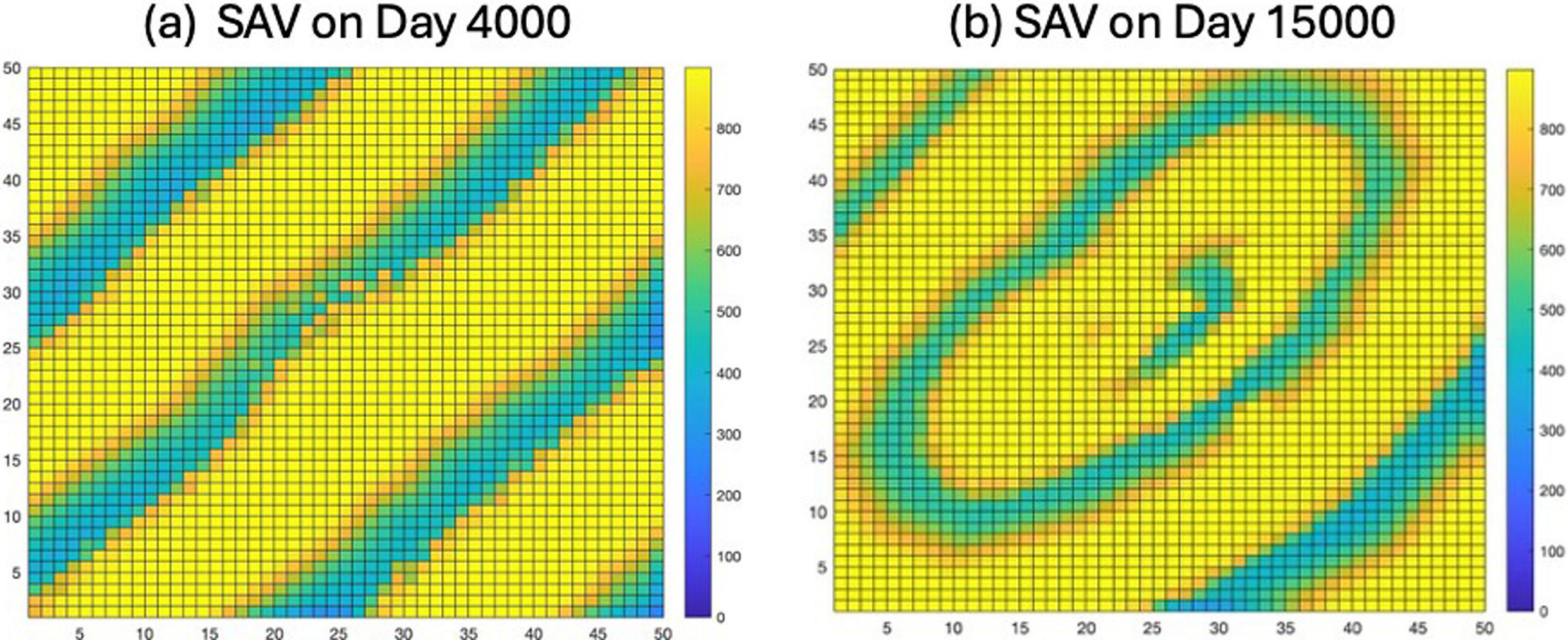
(a) Travelling wave of moving stripes (towards the upper left at about 6 cm day^−1^) of SAV that emerged after about 4000 days into the simulation. (b) The spiralling pattern of circular rings that developed from the initially striped pattern largely between days 13 400 and 14 150.

The results of simulations show a sequence of events that shift the original striped travelling wave to one in which a pattern of rings expands from the centre (figure 2), i.e. a spiralling wave. This was shown consistently in numerous simulations of our model. Here we show only the results for a sequence of events for only one simulation as representative of the general phenomenon of this model in which the striped pattern changes to a spiralling pattern, as shown in figure 2.

To understand how this switch in the pattern developed, we need to look in detail at the dynamics of the system through time. We do this by looking at a few particular time intervals, in which key processes of the transition occur as follows. (i) An initial instability is caused by a stochastic perturbation of addition of adults on day 13 200, which leads to initiation of an instability around day 13 420. (ii) The instability grows and merges with a trailing moving stripe of FAV. (iii) This produces conditions for a second instability starting about day 13 910. (iv) The increase in the larval consumption rate that started on day 13 000 (and is totally independent of the adult-addition-initiated instability) has by this time had feedback effects that led to the start of disappearance of stripes of FAV and weevils 400 days later. (v) The second instability occurs, which is able to expand without encountering other stripes. (vi) A spiral travelling wave of circularly expanding rings of FAV, weevils and SAV develops.

### 3.2. Initial instability

**Figure 3.**
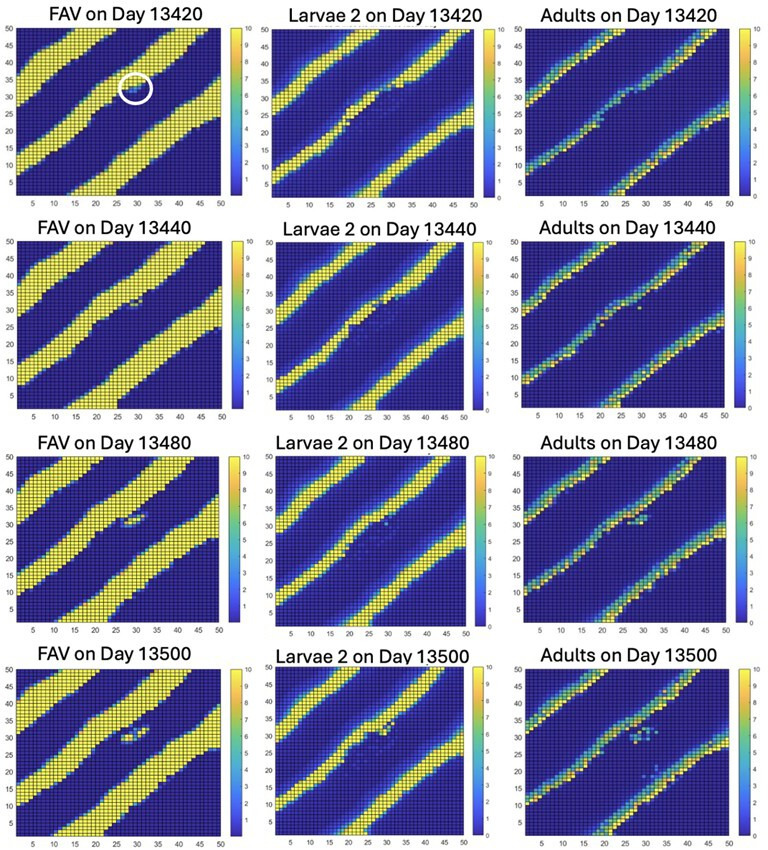
The creation of an instability in the striped pattern, which can be seen to start roughly on day 13 420 (see blue dot within a white circle) and to form a patch of FAV that departs its stripe of origin and grows as a rock–scissors–paper instability during the next 80 days.

Around day 13 420 the start of an instability in the striped pattern is apparent. Figure 3 shows the sequence of events for FAV, adults and larvae. The circled blue dot near the centre of FAV on day 13 420 is the start of this instability. This instability starts because the feedback interactions among the three species following the perturbation on day 13 200 on a small set of pixels led to a region of pixels on which both adult and larval weevils have disappeared or been reduced to very low levels, though some FAV biomass remained. This creates a situation in which the time lag of invasion of new adults (larvae do not move between pixels) and the time lags in days between eggs and L2 larvae and between L2 larvae and adults, allowed FAV in the small region of pixels to grow without hindrance and escape control by weevils. This phenomenon is described in [48] as a rock–scissors–paper instability. By day 13 500, a patch of expanding FAV has detached from the stripe of FAV, and it continues to grow. It is important to emphasize that the external addition of adult weevils is a very small perturbation. Two adult weevils are added every 300 days to pixels in the range (18 ≤ *i* ≤ 32, 18 ≤ *j* ≤ 32) on which FAV biomasses of ≥20 g dW m^−2^ exist. The weakness of this perturbation is evidenced by the striped travelling wave persisting from its emergence by 4000 days to the mid-13 000s.

### 3.3. Merger of unstable patch of FAV with next moving stripe of FAV

**Figure 4.**
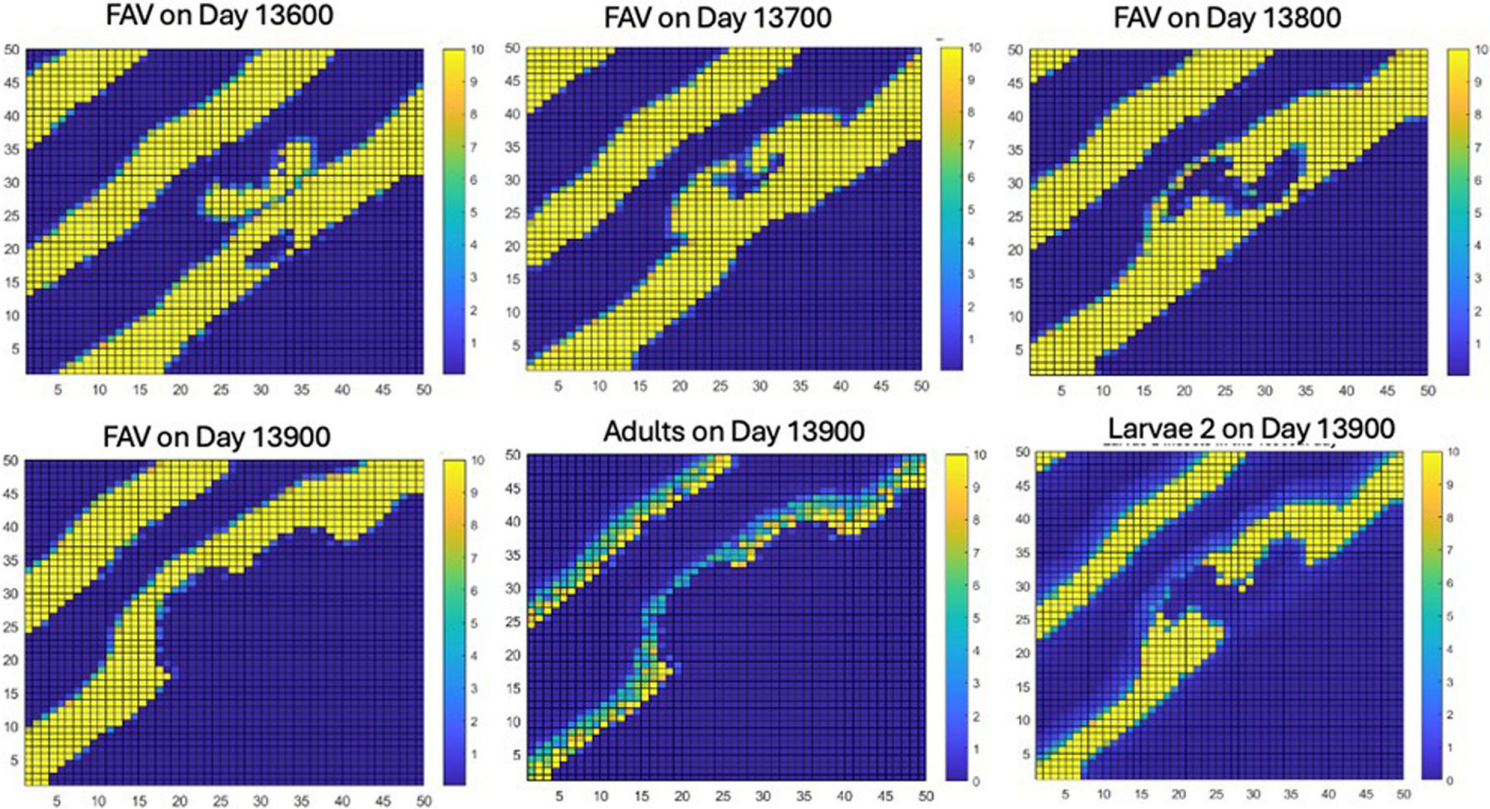
Merger of the rock–scissors–paper instability, shown as a patch of FAV, with the next FAV stripe, which is moving towards the upper right. By day 13 900, this has hollowed out the FAV stripe and those of the adult and larval weevil stripes over an area of pixels. There is no new stripe of FAV moving from the lower right due to increased damage by larval weevils.

The growing patch of escaping FAV eventually merges with the next advancing stripe of FAV. This leads to new interactions between weevils and FAV, and the feedback among these leads to a hollowing out of parts of the FAV stripe where the merger has occurred. Feedback interactions with weevils create a hollowed-out area as well in the adults and L2 weevils (figure 4). In the meantime, the next potential stripe of FAV, which would have occurred in the bottom right corner of figure 4, is not present because of overgrazing of the FAV due to the increased damage done to FAV through consumption rate of weevil larvae, which subsequently also drastically declined in that area.

### 3.4. Formation of the second instability and formation of second spiral wave

**Figure 5.**
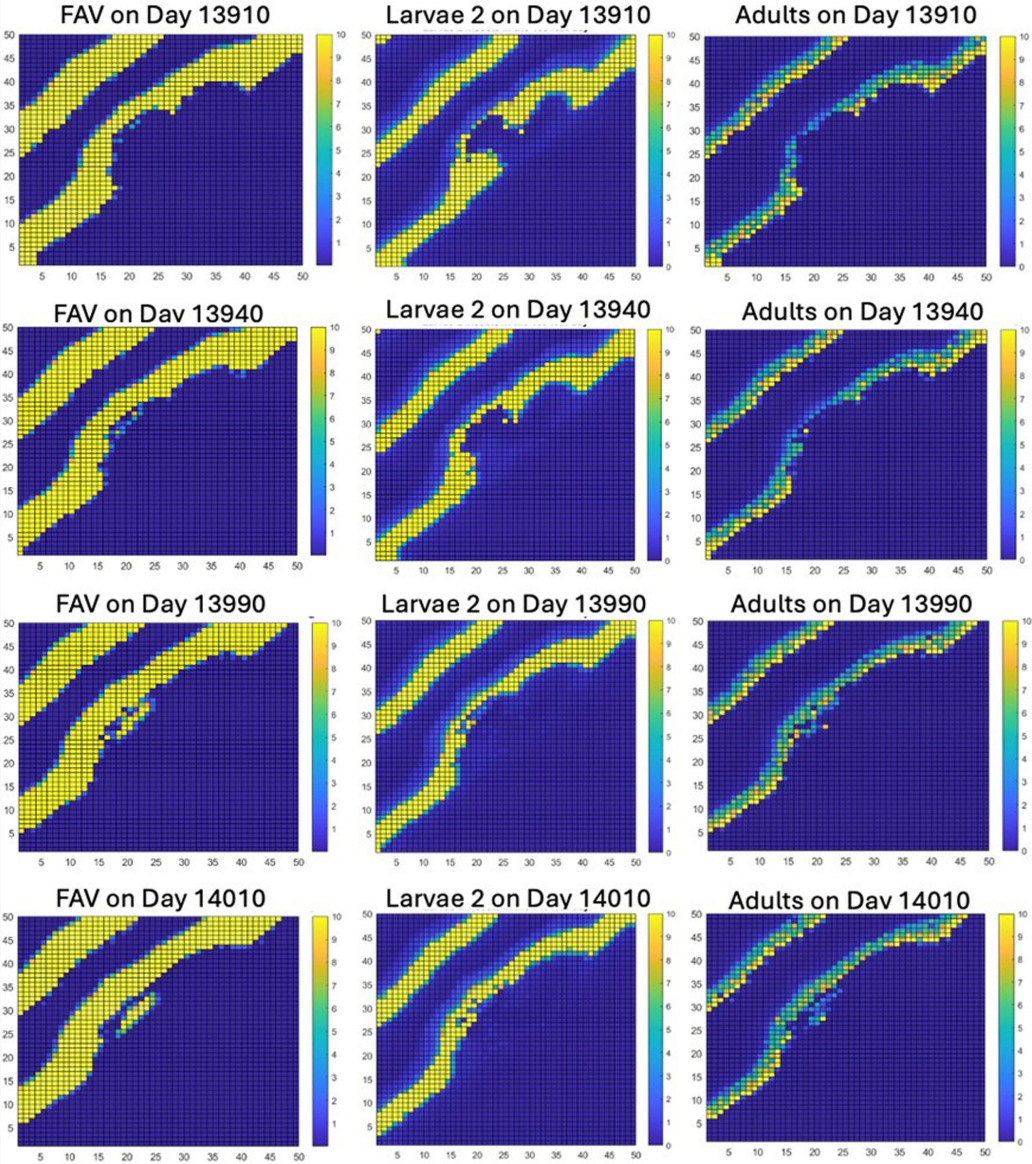
The creation of a second instability in the striped pattern, which can be seen to start approximately on day 13 910 (see blue dots slightly to right of hollowed FAV stripe on day 13 910) and to form a patch that departs its stripe of origin and is shown to continue to grow as a rock–scissors–paper instability during the next 100 days.

The large reduction of both FAV and weevils over a large section of the lattice due to interactions caused by the merger creates conditions for a new instability of the same type that occurred on day 13 420. This instability, starting at roughly day 13 910, then grows into an area on which no further stripes of high densities of FAV or weevils will pass. By day 14 010, this instability had grown to a large patch below the stripe on which it was created (figure 5).

### 3.5. Expansion of instability into an SAV-dominated area

**Figure 6.**
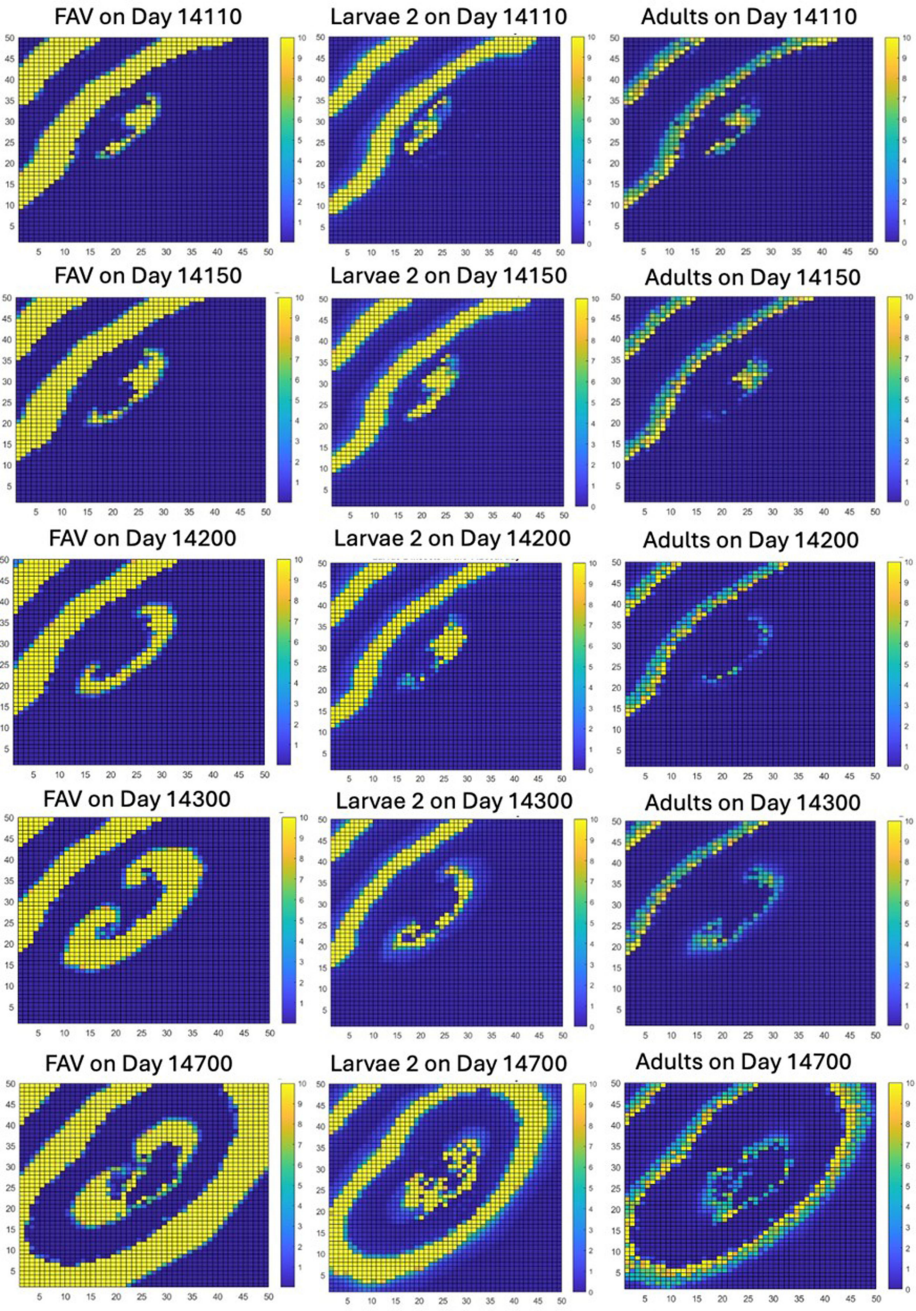
The formation of concentrically expanding rings of rock–scissors–paper spiralling travelling wave.

The instability next expands from its initial point of genesis in the middle of the lattice. Because there are no new visible stripes of FAV or weevils (although these may still exist at densities in the pixels too low to have an effect), the instability spreads out in an SAV-dominated area as a rock–scissors–paper instability, forming a spiral travelling wave (figure 6). The subsequent dynamics of the spiral travelling wave are shown in electronic supplementary material, appendix 3.

### 3.6. Long-term dynamics of the spiral wave

The spiral wave continues for about 1500 days, before breaking into a few smaller spiral waves (electronic supplementary material, appendix 3). However, by about 13 000 days later, the single spiral wave seems to have been restored, though it breaks again into a few smaller spiral waves, which are followed in the simulation only until day 40 000.

### 3.7. Alternative case of transition of plane travelling wave to spiral travelling wave

Here we have shown a particular scenario in which a plane travelling wave transitions to a spiral wave through a pair of instabilities. More generally, any disturbance that leads to the end of the train of stripes of FAV, if accompanied by an instability caused by a stochastic perturbation, can lead to transition of the plane wave to a spiral wave (see electronic supplementary material, appendix 4).

## 4. Discussion

In §2.1 we outlined, in the form of a conceptual model, the characteristics of an ecological system that can lead to the formation of an intransitive (rock–scissors–paper) travelling wave of two competing species and a specialist natural enemy of one of the competitors. We showed this concretely and in detail a well-parametrized model in which these characteristics are incorporated. A plane travelling wave formed, was apparently stable for a long period of time, surviving continual stochastic perturbations in the form of small random numbers of added adult insects, but ultimately ended as a long transient. We described the last days of the long transient’s transition to a spiral travelling wave in detail.

Although we showed here only a single simulation, many additional simulations have been performed using different random number initiators. The results were always similar, though differing in detail, including different parameter sets [48]. A plane striped pattern travelling wave emerges that switches to a spiral wave at some point in time. Differing from the results here, in [48,49] the long transient plane pattern travelling wave switched to a chaotic-appearing pattern. Here we imposed, along with the periodic addition of small numbers of adult weevils, a press perturbation in terms of an increase in the damage to FAV caused by the consumption by the larval weevils, which created conditions by which the plane wave pattern switched to the spiral pattern.

Two instabilities occurred, each a result of stochastic perturbation. The first instability resulted from a small number of adult weevils being externally added to random pixels with FAV in the centre of the lattice on day 13 200. Although small numbers of adults had been added every 300 days from the start of the simulation, this was the first addition of adults to lead to a growing instability. The system was apparently resilient to all the earlier perturbations, but the system’s inherent vulnerability was eventually revealed. The second instability resulted after the merger of the growing patch of FAV with the trailing stripe of SAV. That caused a hollowing out of the FAV, adult and larval weevil stripes over an area of pixels that set up conditions for FAV to escape again, this time into an area of pure SAV. This escape, like the first, was due to the following time lags in the weevil. (i) Only adult weevils were able to move between pixels and only to adjacent pixels. (ii) There are 41 days between the day of egg stage and first day of L2 larva, which can consume FAV biomass. An additional 34 days is required for an initial L2 larva to become an adult. Ironically, it is this time lag that facilitates emergence of the striped travelling wave in the first place, by allowing some partial escape in space of the FAV from the weevil. But now the lag creates conditions for stochastic effects to lead to the end of the striped travelling wave.

Although we have used a specific parametrized model in this study, and the particular resultant dynamics of the end of the long transient has features that seem unique, we argue that its main features are general, and that the results have broad implications for transient dynamics. For one thing, although we used boundary conditions to easily create plane travelling waves, such plane waves have been generated in other models that use periodic conditions [50,51], so they are not an unlikely phenomenon. Our results show that local dispersal is essential to the dynamics of the travelling waves, as in [56]. The results also display the role of stochastic disturbances to long transients, as reviewed by [57]. Also, system dynamics are highly sensitive to the conditions on mobility of the three species. Higher or lower dispersal rates of any species, particularly the natural enemy, would have drastically changed results, in agreement with [58,59]. Our results show that a press perturbation of damage rate by the natural enemy leads to transition of the plane wave to a spiral wave rather than to a chaotic pattern. This agrees with the general results regarding parameter sensitivity of transient dynamic communities [60,61]. Therefore, our model, while parametrized to a specific system, is consistent with general results from more abstract models. We agree with Oster [62], who noted that theory is best developed from the careful study of models developed for specific systems. The dynamics shown here are one specific case of a long transient. But many simulations show that an end will always come. The properties of an intransitive loop, which depend on time delays to lead to spatial structures, doom the structures in the long run when some random disturbance affects those delays.

Equations (2.1) and (2.3) constitute a straightforward, relatively general model for exploitative exploitation between two species and a natural enemy of one. With suitable adjustments, it can be adapted to many different variations of this classic ecological motif. The equations produce a form of self-organization that should be considered, along with the well-known Turing-type self-organization of spatial pattern. For example, dryland ecosystems, in which spiral wave patterns are common (e.g. [47]), have the appearance of resulting from similar intransitive networks. More generally across biology and chemistry, spiral waves emerge in such systems as the cAMP waves of *Dictyostelium discoideum* amoebae, which [63] modelled with three variables, and the Belousov–Zhabotinsky reaction [64].

Our study shows the relationships between several key concepts of ecological theory: spatial self-organization, long transients, intransitive networks, stochastic pulse perturbations, local dispersal and time lags—all in a classic ecological model. It shows how inconspicuous effects occurring at the local scale can lead to macroscopic changes—that is, a butterfly effect. Moreover, our modelling is thoroughly parametrized with empirical data to address a practical environmental issue, which means that its results may have implications beyond theoretical ones.

## Data Availability

The code for the results produced by this paper is available at Dryad [65] and can be freely downloaded. A README file is provided that explains the code. Supplementary material is available online [66].
